# Supplementary material for: Simple and scalable growth of AgCl nanorods by plasma-assisted strain relaxation on flexible polymer substrates
Source: Nat Commun. 2017 Jun 1;8:15650. doi: 10.1038/ncomms15650 (PMC5461508; doi:10.1038/ncomms15650)
Supplement: Supplementary Information — Supplementary Figures, Supplementary Tables and Supplementary References [file ncomms15650-s1.pdf]

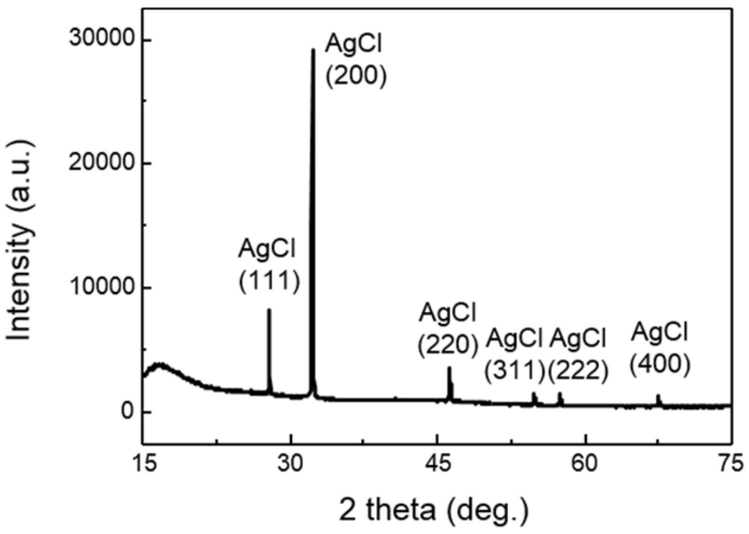

Supplementary Figure 1. X-ray diffraction patterns of the Cl_2 plasma exposed Ag. After Cl_2 plasma treatment, all Ag had reacted completely to produce AgCl compounds.

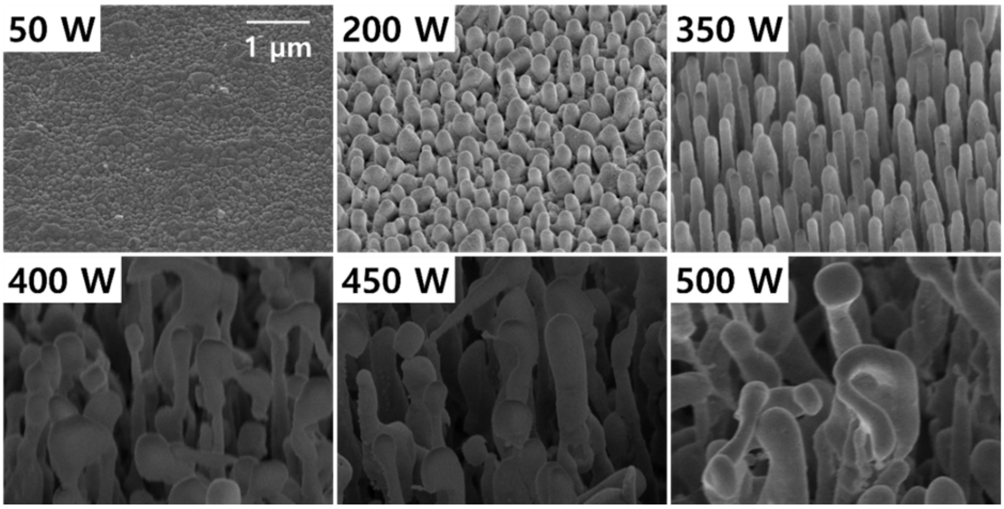

Supplementary Figure 2. Optimization of the plasma power for fabricating AgCl nanorods. 300-nm-thick Ag coated on PI films were exposed to Cl_2 plasma with different plasma power for 45 s at 10 mTorr.

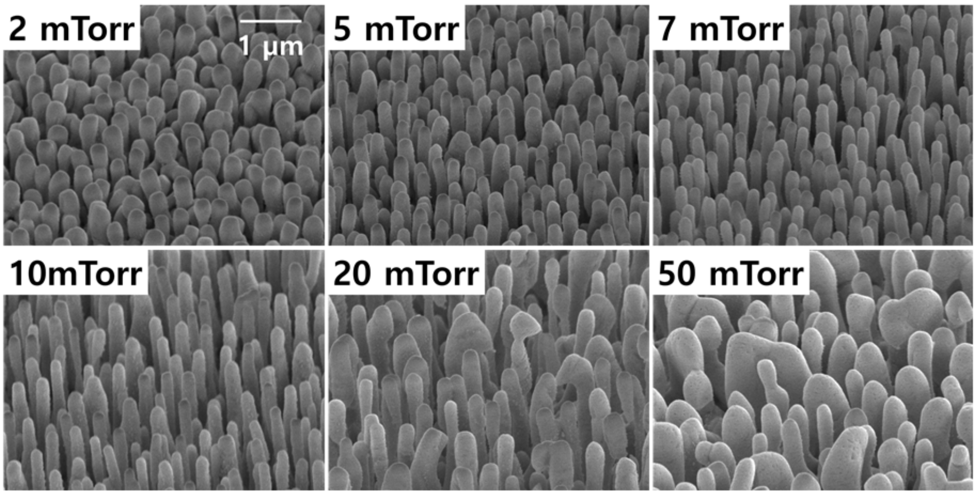

Supplementary Figure 3. Optimization of the plasma process pressure for fabricating AgCl nanorods. 300-nm-thick Ag coated on PI films were exposed to Cl_2 plasma with different process pressures for 45 s at plasma power of 350 W.

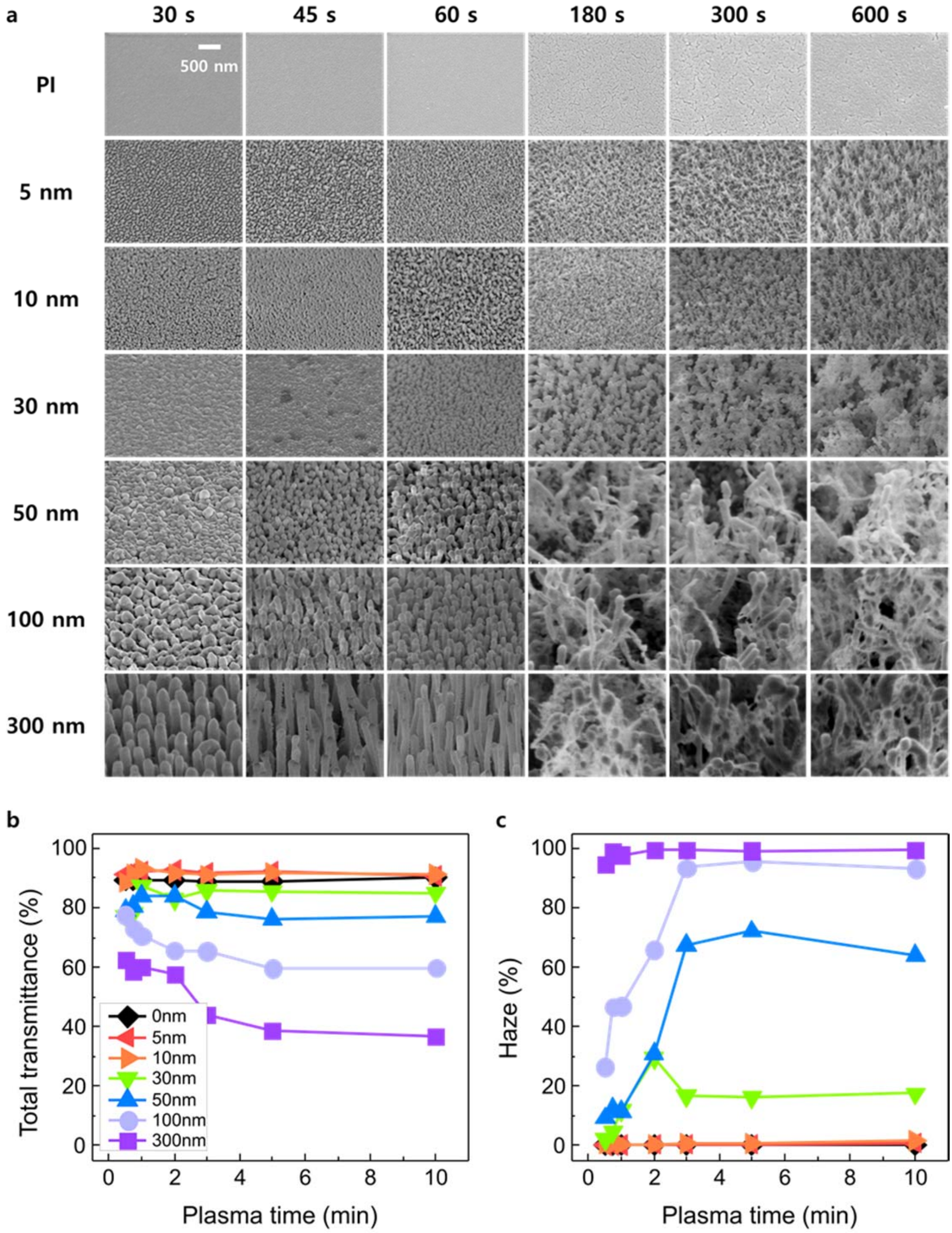

Supplementary Figure 4. Time evolution of Ag film during exposure to Cl_2 plasma. (a) Scanning electron microscopy images, (b) Total optical transmittance and (c) Haze of of AgCl nanostructure as functions of Ag thickness and Cl_2 plasma exposure time.

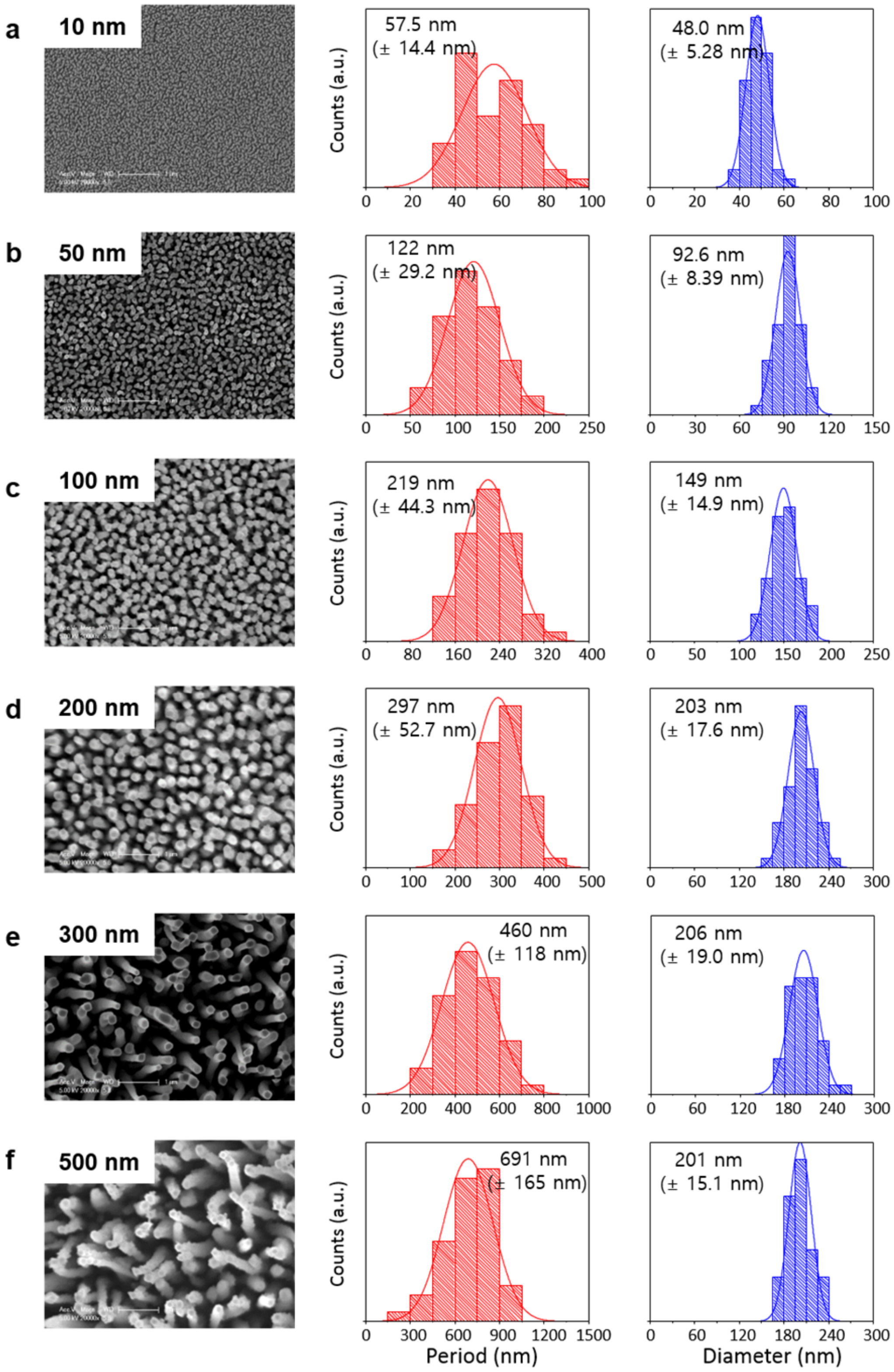

Supplementary Figure 5. Image analysis of AgCl nanorods. AgCl nanorods were fabricated from Ag film with thickness of (a) 10 nm, (b) 50 nm, (c) 100 nm, (d) 200 nm, (e) 300nm, and (f) 500nm. (Left) top view Scanning electron microscopy images of AgCl nanorods and histogram of measured average (middle) period and (right) diameter.

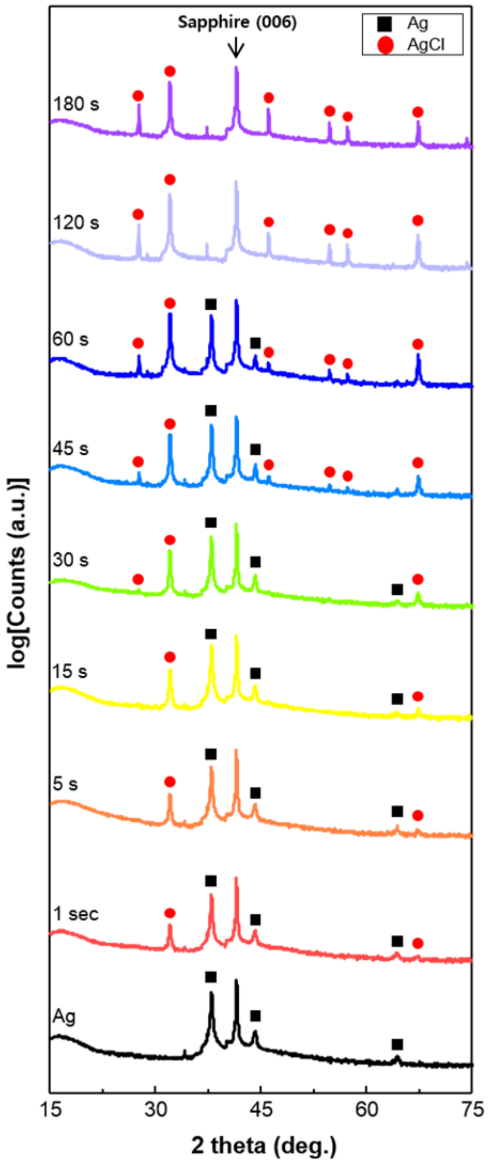

Supplementary Figure 6. XRD pattern of Cl_2-exposed Ag as a function of plasma exposure time on c-sapphire wafer. As on glass, Ag disappeared completely within 120 s of Cl_2 plasma treatment on c-sapphire.

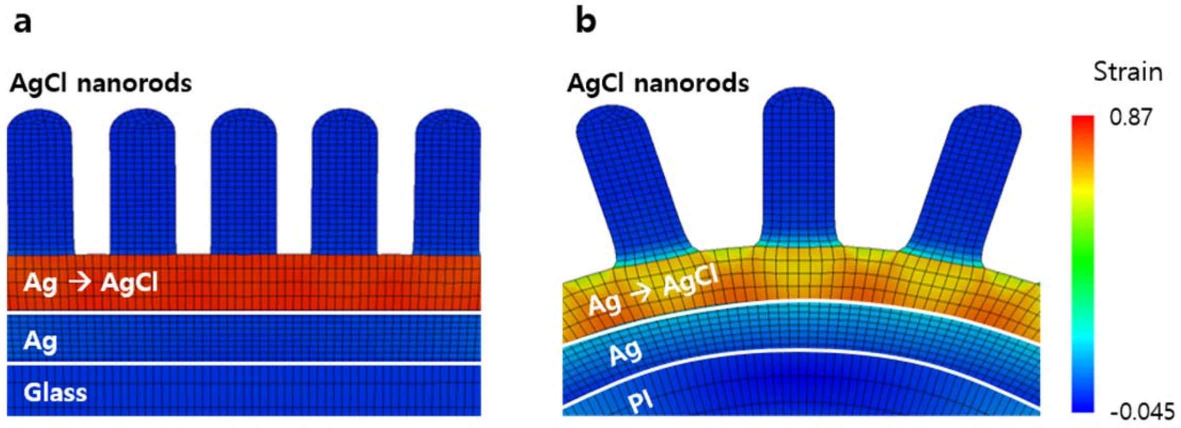

Supplementary Figure 7. Effect of substrate flexibility on strain. Calculated strain under volume expansion from Ag to AgCl on (a) the rigid glass and (b) flexible polyimide film. Volume expansion was assumed to occur at interface of AgCl nanorods with Ag film.

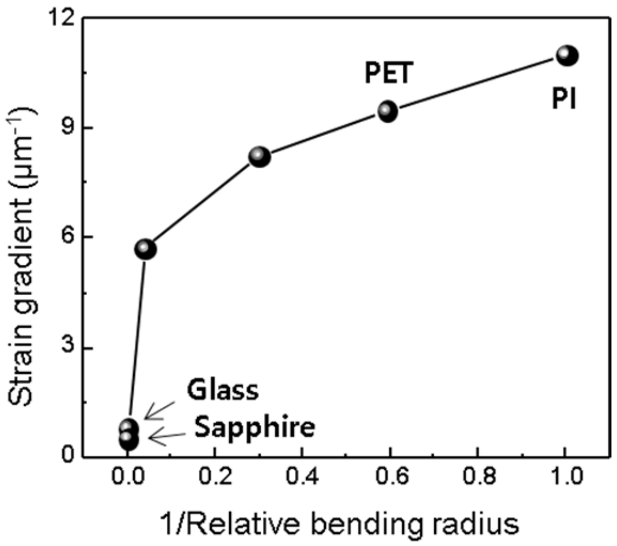

Supplementary Figure 8. Effect of substrate flexibility on strain gradient. Strain gradient at edge of nanorods was calculated as a function of relative bending radius. Relative bending radius was normalized bending radius to that of polyimide (PI) film. PI, polyethylene terephthalate (PET), glass, and sapphire were noted.

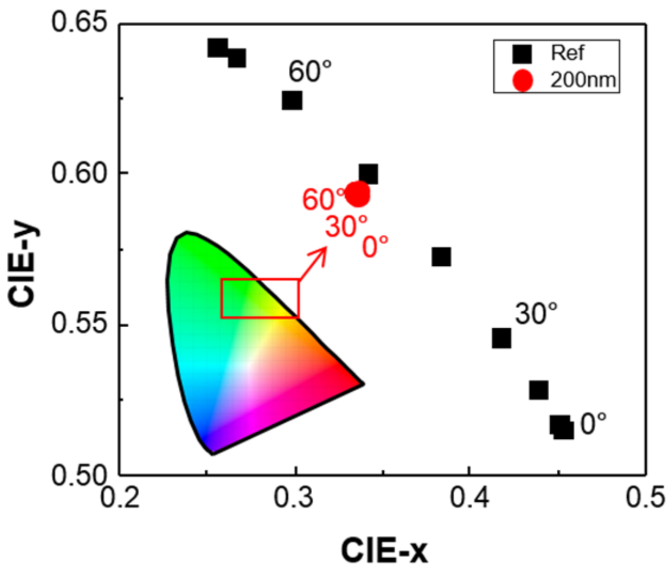

Supplementary Figure 9. Color uniformity of AgCl nanorods implemented Organic light-emitting diodes (OLEDs). CIE color map of luminance as a function of emission angle of reference and AgCl (t_{Ag} = 200 nm) devices. AgCl (t_{Ag} = 200 nm) device showed almost unchanged CIE color coordinates as emission angle increased, whereas reference device showed unstable color stability with emission angle.

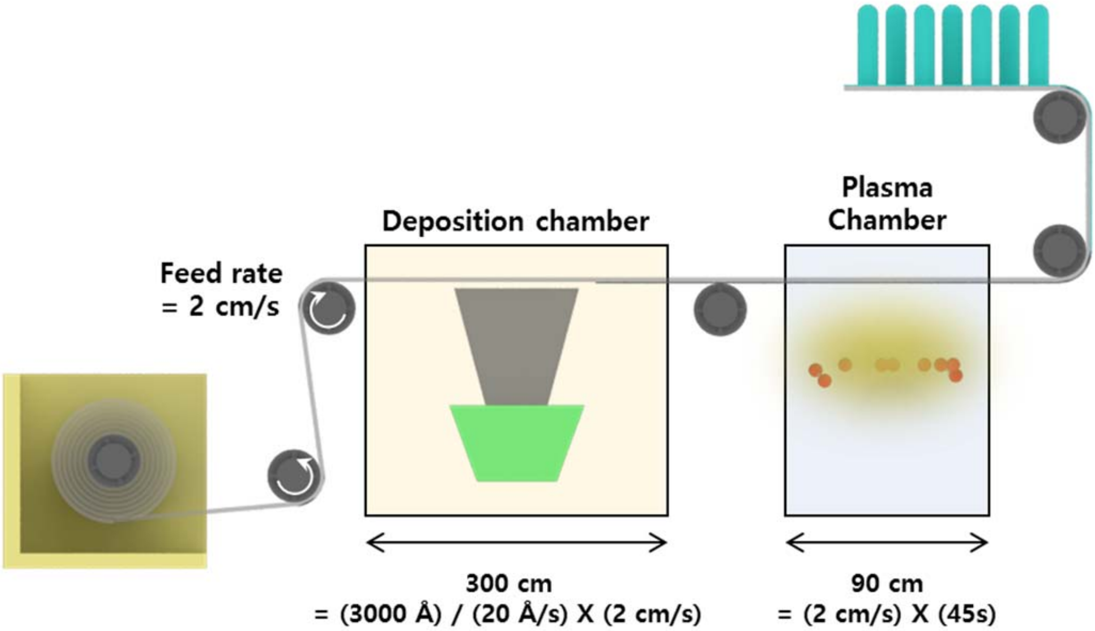

Supplementary Figure 10. Design of roll-to-roll system to fabricate AgCl nanorods. Schematic illustration of roll-to-roll process to produce AgCl nanorods on plastic film.

To confirm the applicability of the method to R2R process, we designed a virtual R2R system (Supplementary Fig. S10). The plasma process time is critical factor for application to R2R process. In Fig. 2, the plasma time of 45 s is enough to get the NRs. Because the deposition rate of Ag layer did not have a significant effect on morphology or growth rate of AgCl NRs (Supplementary Fig. S11), the deposition rate was set to be 20 Å s^{-1}. When the plasma chamber was 90 cm long and the Ag deposition chamber was 300 cm long, the feed rate of R2R process was 1.2 m min^{-1}, which is compatible with a commercial process.

$$\text{Length of plasma chamber} = (\text{Feed rate})(\text{Plasma time}) = (1.2\text{m min}^{-1})(45\text{ s}) = 90\text{ cm}$$

To deposit a 300-nm-thick Ag layer at a rate of 20 Å/s, the length of Ag deposition chamber should be 300 cm:

$$\text{Length of deposition chamber} = \frac{(\text{Thickness of Ag})}{(\text{Deposition rate})} \times (\text{Feed rate})$$

$$= \frac{3000\text{ Å}}{20\text{Å s}^{-1}} \times (1.2\text{ m min}^{-1}) \quad = 300\text{ cm}$$

Those feed rate and chamber size are compatible with common R2R systems, and can produce AgCl NRs on polymer film at 2 cm s^{-1}.

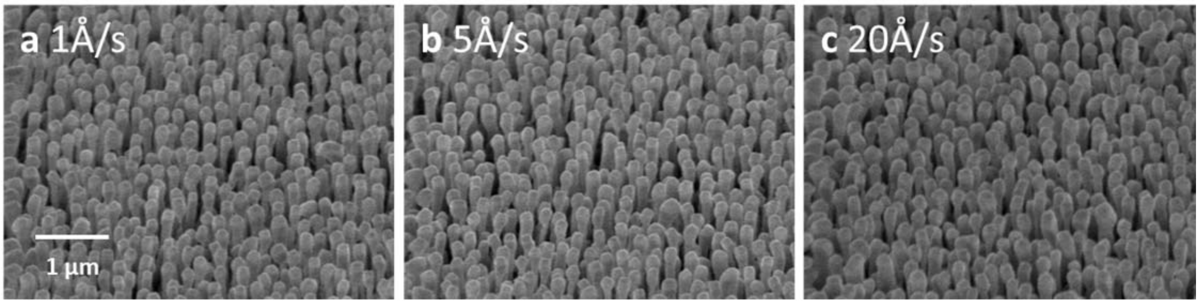

Supplementary Figure 11. Effect of deposition rate of Ag on AgCl nanorods. Scanning electron microscopy images of AgCl nanorods as a function of Ag deposition rate. Deposition rate did not have a significant effect on the morphology of nanorods.

Supplementary Table 1. Elastic properties and thickness of substrates. Relative radius of curvature was calculated using Stoney equation, then normalized to that of polyimide film. Stress-thickness products of film ($\sigma_f t_f$) were assumed to be constant on each substrate.

Substrates	Young's modulus (E_s) [GPa]	Possion's ratio (υ_s)	Substrate thickness (t_s) [um]	Relative radius of curvature
PI	2.5	0.4	100	1
PET	2.5	0.4	130	1.69
PC	2.5	0.4	500	25
Glass	74	0.3	700	1243.2
Sapphire	345	0.3	500	2957

The relationship between radius (r) of curvature of the substrate and film stress (σ_f) is given by the Stoney equation

$$r = \frac{E_s t_s^2}{6(1 - \upsilon_s)\sigma_f t_f}$$

where r is the radius of curvature, σ_f is the film stress, E_s is the Young's modulus of the substrate, υ_s is its Poisson's ratio, t_s is the substrate thickness, and t_f is the film thickness. The calculated r values of substrates were normalized to that of PI. Normalized r were $r \equiv 1$ for PI, r = 1.69 for PET, r = 25 for PC, r = 1243 for glass and r = 2957 for sapphire (Supplementary Table S1). Because the polymer films (PI, PET, and PC) had similar elastic properties (E_s = 2.5 GPa, υ_s = 0.4), their radius of curvature was only proportional to square of t_s. Thus, r was the smallest on 100-µm-thick PI film, and increased in the sequence of 130-µm-thick PET and 500-µm-thick PC film (Fig. 6d). The 700-µm-thick glass had larger E_s = 74 GPa than the polymers, so its r was expected to 1,000 times larger than that of PI film; i.e., the glass showed almost no flexion during volume expansion.

Supplementary Table 2. Properties used in calculation of the atomic flux.

Symbol	Property	Value	Unit	Note
C	Concentration	$5.85\text{x}10^{22}$	$\#/\text{cm}^3$	Intrinsic property of Ag
Ω	Atomic volume	$1.26\text{x}10^{-23}$	cm^3	Intrinsic property of Ag
E	Young's modulus	$8.30\text{x}10^{11}$	Pa	Intrinsic property of Ag
$k_B T$	Energy	$4.11\text{x}10^{-21}$	J	Thermal energy at RT
∂x	Change in x	$2.5\text{x}10^{-6}$	cm	Set to be 25 nm
$\partial \varepsilon$	Change in ε	0.275	-	Change in strain with 20 nm change in x at the edge of the nanorods

The atomic flux under the strain can be described as

$$J = J_{\text{Concentration}} + J_{\text{strain}} = -D\frac{\partial C}{\partial x} - D\frac{C\Omega E}{k_B T}\frac{\partial \varepsilon}{\partial x} = -D\left(\frac{\partial C}{\partial x} + \frac{C\Omega E}{k_B T}\frac{\partial \varepsilon}{\partial x}\right)$$

where C is the atomic concentration, Ω is the atomic volume, D is the local diffusion coefficient, E is the Young's modulus, k_B is Boltzmann`s constant, T is absolute temperature, and ε is the strain. To compare the concentration gradient and strain gradient, the gradients were calculated numerically with the properties in Table S2. The change in *x*was set to be *dx* = 50 nm.

$$\frac{\partial C}{\partial x} = \frac{5.85\times10^{22}\ \text{atoms cm}^{-3}}{2.5\times10^{-6}\ \text{cm}^{-1}} = 2.34\times10^{28}\text{atoms cm}^{-4}$$

$$\frac{C\Omega E}{k_B T}\frac{\partial \varepsilon}{\partial x} = \frac{(5.85\times10^{22}\ \text{atoms cm}^{-3})\cdot(1.26\times10^{-23}\ \text{cm}^3)\cdot(8.3\times10^{5}\ \text{J cm}^{-3})}{(4.11\times10^{-21}\text{J})}\frac{0.275}{2.5\times10^{-6}\ \text{cm}^{-1}} = 1.64\times10^{31}\text{atoms cm}^{-4}$$

The strain gradient term was about 700 times larger than concentration gradient, so atomic flux follows the strain-induced diffusion.

Supplementary Table 3. Growth temperature and rate of various inorganic nanorods

Growth Method	Materials	Growth Temperature [°C]	Growth Rate [nm min^{-1}]	Ref.
Hydrothermal	ZnO	90	0.6	1
	ZnO	60	1	2
	ZnO	95	6.25	3
	ZnO	70	2.9	4
	ZnO	79	11.4	5
	ZnO	90	5	6
	ZnO	90	6	7
	ZnO	100	2.8	8
	TiO_2	140	3.7	9
	TiO_2	150	1.2	10
	TiO_2	90	17.1	11
	TiO_2	150	5.6	12
	TiO_2	170	0.23	13
	TiO_2	190	23	14
	TiO_2	210	10	15
	TiO_2	200	2.08	16
	Ta_2O_5	240	0.41	17
	WO_3	170	7.5	18
	WO_3	180	6.25	19
	Nb_2O_5	150	4	20
	Fe_2O_3	60	1.3	21
	CoO	120	17	22
Vapor-liquid-solid	ITO	300	30	23
	ITO	800	16.3	24
	ZnO	990	100	25
	ZnO	820	230	26
	ZnO	750	15	27
	NiO	550	30	28
	MgO	925	1.7	29

	SnO_2	750	5	27
	In_2O_3	750	15	27
	In_2O_3	750	-	30
	MgO	750	17	27
Chemical Vapor Deposition	ZnO	650	90	31
	TiO_2	550	-	32
	WO_3	500	61	33
	WO_3	500	108	34
	CuO	600	900	35
	SnO_2	760	55.5	36
	Ga_2O_3	1000	120	37
Thermal oxidation	ZnO	500	33.3	38
	CuO	400	13.8	39
	CuO	400	5.4	40
	CuO	400	14	41
	CuO	600	9.7	42
	CuO	400	11.1	43
	CuO	400	20	44
	$W_{18}O_{49}$	700	12	45
**AgCl**	**This work**	**~ RT**	**2000**	

Supplementary References:

1. Cheng J. J., Nicaise S. M., Berggren K. K., Gradečak S. Dimensional tailoring of hydrothermally grown zinc oxide nanowire arrays. *Nano Lett.* **16**, 753-759 (2015).
2. Joo J., Chow B. Y., Prakash M., Boyden E. S., Jacobson J. M. Face-selective electrostatic control of hydrothermal zinc oxide nanowire synthesis. *Nat. Mater.* **10**, 596-601 (2011).
3. Park G. C.*, et al.* Hydrothermally grown In-doped ZnO nanorods on p-GaN films for color-tunable heterojunction light-emitting-diodes. *Sci. Rep.* **5**, 10410 (2015).
4. Lee J. M., No Y.-S., Kim S., Park H.-G., Park W. I. Strong interactive growth behaviours in solution-phase synthesis of three-dimensional metal oxide nanostructures. *Nat. commun.* **6**, 6325 (2015).
5. Watanabe K.*, et al.* Arbitrary cross-section SEM-cathodoluminescence imaging of growth sectors and local carrier concentrations within micro-sampled semiconductor nanorods. *Nat. commun.* **7**, 10609 (2016).
6. Kim B. H., Kwon J. W. Metal catalyst for low-temperature growth of controlled zinc oxide nanowires on arbitrary substrates. *Sci. Rep.* **4**, 4379 (2014).
7. Consonni V.*, et al.* Selective area growth of well-ordered ZnO nanowire arrays with controllable polarity. *ACS nano* **8**, 4761-4770 (2014).
8. Yue H. Y.*, et al.* ZnO nanowire arrays on 3D hierachical graphene foam: biomarker detection of Parkinson's disease. *ACS nano* **8**, 1639-1646 (2014).
9. Ye M., Liu H. Y., Lin C., Lin Z. Hierarchical Rutile TiO2 Flower Cluster-Based High Efficiency Dye-Sensitized Solar Cells via Direct Hydrothermal Growth on Conducting Substrates. *Small* **9**, 312-321 (2013).
10. Huang H.*, et al.* Hydrothermal Growth of TiO2 Nanorod Arrays and In Situ Conversion to Nanotube Arrays for Highly Efficient Quantum Dot-Sensitized Solar Cells. *Small* **9**, 3153-3160 (2013).
11. Yang T.*, et al.* Position-controlled hydrothermal growth of periodic individual ZnO nanorod arrays on indium tin oxide substrate. *J. Phys. Chem. C* **118**, 20613-20619 (2014).
12. Berhe S. A., Nag S., Molinets Z., Youngblood W. J. Influence of Seeding and Bath Conditions in Hydrothermal Growth of Very Thin (～ 20 nm) Single-Crystalline Rutile TiO2 Nanorod Films. *ACS Appl. Mater. Interfaces* **5**, 1181-1185 (2013).

13. Chen J., Yang H. B., Miao J., Wang H.-Y., Liu B. Thermodynamically driven one-dimensional evolution of anatase TiO2 nanorods: one-step hydrothermal synthesis for emerging intrinsic superiority of dimensionality. *J. Am. Chem. Soc.* **136**, 15310-15318 (2014).
14. Wu W. Q., Huang F., Chen D., Cheng Y. B., Caruso R. A. Thin Films of Dendritic Anatase Titania Nanowires Enable Effective Hole-Blocking and Efficient Light-Harvesting for High-Performance Mesoscopic Perovskite Solar Cells. *Adv. Funct. Mater.* **25**, 3264-3272 (2015).
15. Resasco J., Dasgupta N. P., Rosell J. R., Guo J., Yang P. Uniform doping of metal oxide nanowires using solid state diffusion. *J. Am. Chem. Soc.* **136**, 10521-10526 (2014).
16. Wang C.-C., Hsueh Y.-C., Su C.-Y., Kei C.-C., Perng T.-P. Deposition of uniform Pt nanoparticles with controllable size on TiO2-based nanowires by atomic layer deposition and their photocatalytic properties. *Nanotechnology* **26**, 254002 (2015).
17. Su Z., Wang L., Grigorescu S., Lee K., Schmuki P. Hydrothermal growth of highly oriented single crystalline Ta 2 O 5 nanorod arrays and their conversion to Ta 3 N 5 for efficient solar driven water splitting. *Chem. Commun.* **50**, 15561-15564 (2014).
18. Zheng F., Lu H., Guo M., Zhang M., Zhen Q. Hydrothermal preparation of WO 3 nanorod array and ZnO nanosheet array composite structures on FTO substrates with enhanced photocatalytic properties. *Journal of Materials Chemistry C* **3**, 7612-7620 (2015).
19. Zheng F.*, et al.* Hydrothermal preparation, growth mechanism and supercapacitive properties of WO 3 nanorod arrays grown directly on a Cu substrate. *CrystEngComm* **18**, 3891-3904 (2016).
20. He J.*, et al.* Hydrothermal growth and optical properties of Nb 2 O 5 nanorod arrays. *Journal of Materials Chemistry C* **2**, 8185-8190 (2014).
21. Kong D.*, et al.* Seed-assisted growth of α-Fe 2 O 3 nanorod arrays on reduced graphene oxide: a superior anode for high-performance Li-ion and Na-ion batteries. *J. Mater. Chem. A* **4**, 11800-11811 (2016).
22. Cao L.*, et al.* Vertically aligned cobalt oxide nanowires on graphene networks for high-performance lithium storage. *Nanotechnology* **25**, 445704 (2014).
23. Yu H. K., Lee J.-L. Growth mechanism of metal-oxide nanowires synthesized by electron beam evaporation: A self-catalytic vapor-liquid-solid process. *Sci. Rep.* **4**, 6589 (2014).

24. Shen Y., *et al.* Epitaxy-Enabled Vapor–Liquid–Solid Growth of Tin-Doped Indium Oxide Nanowires with Controlled Orientations. *Nano Lett.* **14**, 4342-4351 (2014).
25. Sallet V., Sartel C., Vilar C., Lusson A., Galtier P. Opposite crystal polarities observed in spontaneous and vapour-liquid-solid grown ZnO nanowires. *Appl. Phys. Lett.* **102**, 182103 (2013).
26. Cheng G., *et al.* Large anelasticity and associated energy dissipation in single-crystalline nanowires. *Nat. Nanotech.* **10**, 687-691 (2015).
27. Klamchuen A., *et al.* Rational Concept for Designing Vapor–Liquid–Solid Growth of Single Crystalline Metal Oxide Nanowires. *Nano Lett.* **15**, 6406-6412 (2015).
28. Nagashima K., *et al.* Tailoring Nucleation at Two Interfaces Enables Single Crystalline NiO Nanowires via Vapor–Liquid–Solid Route. *ACS Appl. Mater. Interfaces* **8**, 27892-27899 (2016).
29. Li L., Zhang X., Li L., Zhai X., Zeng C. Magnetoresistance of single-crystalline La 0.67 Sr 0.33 MnO 3/MgO nanorod arrays. *Solid State Commun.* **171**, 46-49 (2013).
30. Domènech-Gil G., *et al.* Gas sensors based on individual indium oxide nanowire. *Sensors and Actuators B: Chemical* **238**, 447-454 (2017).
31. Xu L., *et al.* Catalyst-free, selective growth of ZnO nanowires on SiO2 by chemical vapor deposition for transfer-free fabrication of UV photodetectors. *ACS Appl. Mater. Interfaces* **7**, 20264-20271 (2015).
32. Chen C., *et al.* Growth and characterization of well-aligned densely-packed rutile TiO2 nanocrystals on sapphire substrates via metal–organic chemical vapor deposition. *Nanotechnology* **19**, 075611 (2008).
33. Annanouch F. E., *et al.* Aerosol-assisted CVD-grown WO3 nanoneedles decorated with copper oxide nanoparticles for the selective and humidity-resilient detection of H2S. *ACS Appl. Mater. Interfaces* **7**, 6842-6851 (2015).
34. Annanouch F. E., *et al.* Aerosol-assisted CVD-grown PdO nanoparticle-decorated tungsten oxide nanoneedles extremely sensitive and selective to hydrogen. *ACS Appl. Mater. Interfaces* **8**, 10413-10421 (2016).
35. Lugo-Ruelas M., *et al.* Synthesis, microstructural characterization and optical properties of CuO nanorods and nanowires obtained by aerosol assisted CVD. *Journal of Alloys and Compounds* **643**, S46-S50 (2015).

36. Deng K., Lu H., Shi Z., Liu Q., Li L. Flexible Three-Dimensional SnO2 Nanowire Arrays: Atomic Layer Deposition-Assisted Synthesis, Excellent Photodetectors, and Field Emitters. *ACS Appl. Mater. Interfaces* **5**, 7845-7851 (2013).

37. Hosein I. D., Hegde M., Jones P. D., Chirmanov V., Radovanovic P. V. Evolution of the faceting, morphology and aspect ratio of gallium oxide nanowires grown by vapor–solid deposition. *J. Cryst. Growth* **396**, 24-32 (2014).

38. Zhao C.*, et al.* Large-scale synthesis of bicrystalline ZnO nanowire arrays by thermal oxidation of zinc film: growth mechanism and high-performance field emission. *Cryst. Growth Des.* **13**, 2897-2905 (2013).

39. Zhang Q.*, et al.* Facile large-scale synthesis of vertically aligned CuO nanowires on nickel foam: Growth mechanism and remarkable electrochemical performance. *J. Mater. Chem. A* **2**, 3865-3874 (2014).

40. Rackauskas S.*, et al.* In situ study of noncatalytic metal oxide nanowire growth. *Nano Lett.* **14**, 5810-5813 (2014).

41. Wang J.*, et al.* Three-dimensional hierarchical Co 3 O 4/CuO nanowire heterostructure arrays on nickel foam for high-performance lithium ion batteries. *Nano Energy* **6**, 19-26 (2014).

42. Li A., Song H., Zhou J., Chen X., Liu S. CuO nanowire growth on Cu 2 O by in situ thermal oxidation in air. *CrystEngComm* **15**, 8559-8564 (2013).

43. Tang C.*, et al.* Enhanced adhesion and field emission of CuO nanowires synthesized by simply modified thermal oxidation technique. *Nanotechnology* **27**, 395605 (2016).

44. Kargar A.*, et al.* ZnO/CuO heterojunction branched nanowires for photoelectrochemical hydrogen generation. *ACS nano* **7**, 11112-11120 (2013).

45. Zhang Z.*, et al.* Atomic-Scale Observation of Vapor–Solid Nanowire Growth via Oscillatory Mass Transport. *ACS nano* **10**, 763-769 (2015).
